# Hundred Years of Environmental Change and Phytoplankton Ecophysiological Variability Archived in Coastal Sediments

**DOI:** 10.1371/journal.pone.0061184

**Published:** 2013-04-11

**Authors:** Sofia Ribeiro, Terje Berge, Nina Lundholm, Marianne Ellegaard

**Affiliations:** 1 Department of Biology, Faculty of Science, University of Copenhagen, Copenhagen, Denmark; 2 Marine Biological Section, Department of Biology, University of Copenhagen, Helsingør, Denmark; 3 Natural History Museum of Denmark, University of Copenhagen, Copenhagen, Denmark; Swedish University of Agricultural Sciences, Sweden

## Abstract

Marine protist species have been used for several decades as environmental indicators under the assumption that their ecological requirements have remained more or less stable through time. However, a growing body of evidence suggests that marine protists, including several phytoplankton species, are in fact highly diverse and may quickly respond to changes in the environment. Predicting how future climate will impact phytoplankton populations is important, but this task has been challenged by a lack of time-series of ecophysiological parameters at time-scales relevant for climate studies (i.e. at least decadal). Here, we report on ecophysiological variability in a marine dinoflagellate over a 100-year period of well-documented environmental change, by using the sedimentary archive of living cysts from a Scandinavian fjord (Koljö Fjord, Sweden). During the past century, Koljö Fjord has experienced important changes in salinity linked to the North Atlantic Oscillation (NAO). We revived resting cysts of *Pentapharsodinium dalei* preserved in the fjord sediments and determined growth rates for 18 strains obtained from 3 sediment core layers at salinity 15 and 30, which represent extreme sea-surface conditions during periods of predominantly negative and positive NAO phases, respectively. Upper pH tolerance limits for growth were also tested. In general, *P. dalei* grew at a higher rate in salinity 30 than 15 for all layers, but there were significant differences among strains. When accounting for inter-strain variability, cyst age had no effect on growth performance or upper pH tolerance limits for this species, indicating a stable growth response over the 100-year period in spite of environmental fluctuations. Our findings give some support for the use of morphospecies in environmental studies, particularly at decadal to century scales. Furthermore, the high intra-specific variability found down to sediment layers dated as ca. 50 years-old indicates that cyst-beds of *P. dalei* are repositories of ecophysiological diversity.

## Introduction

Our knowledge of past environments and climate change throughout Earth's history depends on proxy and modelling data, as instrumental recording only began by the end of the 1800 s and for most parameters only within the past few decades. Environmental reconstructions using biological proxies make use of present-day species ecologies to infer past conditions. It is assumed that species distributions and abundances reflect their response to environmental gradients, and that their environmental optima have remained more or less stable over time. In the marine realm, protists such as foraminifera, coccolithophores, diatoms, and dinoflagellate are widely used as climate proxies, due to their high numbers and rich fossil record. They are identified on the basis of their morphology, as morphospecies. Marine protist species have a long evolutionary history, short generation times, huge population sizes, and a large potential to disperse. This has led some authors to argue that protist species are ubiquitous and present little phenotypic variation [1], [2]. In contrast with this view, increasing evidence from molecular and ecophysiological studies [3], [4], [5], [6] suggest that morphospecies of marine protists are in fact highly differentiated. Several laboratory studies, e.g. [7], [8], [9] have revealed large intraspecific variation for key ecophysiological properties. Therefore, the use of morphospecies in environmental research may disregard important diversity and the potential for natural populations to respond rapidly to changing environmental conditions [10].

The response of some present-day phytoplankton species to projected future climate scenarios has been tested under controlled laboratory conditions [8], [9], [11], [12], [13]. Although this approach is suitable for detecting phenotypic variability and immediate responses (e.g. acclimation), it is of limited value to assess evolutionary responses, because it does not allow gradual adaptation to occur over many generations. In order to improve such predictions, it is important to understand temporal intraspecific variability, at timescales relevant for climate studies (i.e. decades rather than seasons). One possible approach is to take advantage of the fact that several phytoplankton groups (notably diatoms and dinoflagellates) form resting stages as part of their life-cycles. These resting stages are deposited in marine sediments and can remain viable for at least a century [14], [15], [16]. Resting stages are physiologically dormant and can be viewed as “time capsules”, as they allow for the preservation of biological material through time. The germination of resting stages formed in the past provides a novel possibility to directly test the response of past living populations to inferred changes in the environment at a decadal to century scale.

Koljö Fjord, a sill fjord located on the west coast of Sweden, offers exceptional conditions for such temporal studies. A combination of very limited oxygen supply, virtually no bioturbation, and minimum tidal activity has built up a natural archive of fine and undisturbed sediments [17], [18]. A relatively long series of historical hydrographic data have been collected in Koljö Fjord since the 1930's [18], [19]. Hydrographic conditions in the fjord are influenced by the North Atlantic Oscillation (NAO). During negative phases of the NAO, cold winters are frequent in the area, as well as a wind regime that enhances upwelling offshore, resulting in strong water-column stratification and bottom oxygen depletion. In contrary, positive phases of the NAO generally lead to a well-mixed water-column and higher bottom oxygen levels in this fjord [18]. During the past century, the NAO has oscillated from a predominantly positive phase (from ca. 1900–1930) to a negative phase (from ca. 1930–1970), returning to the positive phase of today after the 1970's–1980's [18], [19], [20]. These shifts in NAO conditions are reflected in the sedimentary record of the fjord. During the period 1930–1970, bottom salinities were typically between 28.5–31 [18], and the water column stratified, with surface salinities reaching 16 [21]. In contrast, during the predominantly positive NAO period after 1970–1980, deposited fjord sediments are non-laminated, indicating a mixed water-column, and bottom salinities typically varied between 26–29 [18]. Hence, phytoplankton species dwelling in the surface waters of the fjord have, over the past century, experienced extremes of salinity ranging roughly from 15 to 30.

The target species for this study, *Pentapharsodinium dalei*, is a small thecate dinoflagellate common in shallow marine environments north of the North Atlantic Current [22]. Cysts of this species are often a dominant component of polar and cold-temperate dinoflagellate cyst assemblages [20], [22], [23], [24], [25]. In the North Atlantic and adjacent seas, *P. dalei* cysts represent >25% of assemblages in areas with summer salinities between 20–29 [22]. This species has been found as part of the spring bloom in temperate waters [23], [26], whereas in Arctic fjords it is most abundant during late summer-when stratified high productive conditions prevail [27]. In Koljö Fjord, the cyst record of *P. dalei* indicates that it has been more abundant during the negative phase of the NAO (ca. 1930–1970) and decreased markedly during positive phases of the NAO (before 1930 and after 1970) [20].

Our aim was to investigate the ecophysiological response of *Pentapharsodinium dalei* through the past ca. 100 years, a period spanning well-documented salinity shifts in the fjord. For that, we determined the growth rates of 18 revived strains from three discrete sediment layers deposited during 1) the positive NAO phase before 1930; 2) the negative NAO phase between 1930–1970 and, 3) the recent positive phase. The strains were grown at high (30) and low salinity (15), simulating NAO+ and NAO- extreme scenarios, respectively. Our hypothesis was that, if the salinity shifts had represented a strong selective pressure and natural selection had acted at a decadal scale, the recent and oldest strains (NAO+; higher surface water salinity) would have higher fitness (i.e. growth rates) in the high salinity treatment, while the strains collected from the intermediate layer (NAO-; lower surface water salinity) would have higher growth rates in the low salinity treatment. We further monitored pH levels during the experiment, and estimated the upper pH tolerance limit for growth of each strain, to further characterise intraspecific variability at the ecophysiological level [28], [29], [30].

This study represents the first attempt to use the living sedimentary record of phytoplankton resting stages to study adaptation to environmental change at the ecophysiological level. Our results highlight the relevance of intraspecific diversity in determining the tolerance of a population to environmental change, and further emphasize the role of resting stage banks as depositories of biodiversity.

## Materials and Methods

Five sediment cores were retrieved from Koljö Fjord with a modified micro-Kullenberg piston-corer from 45 m water-depth at 58°13 N, 11°34 E in April 2006. All cores were X-rayed while intact and ^210^Pb, ^226^Ra and ^137^Cs activities were analysed via γ-gammaspectrometry for K4, a 73 cm-long core. A combined CRS-CIC model was applied to establish the chronology of the sediments [31]. The other cores were correlated with K4 based on easily discernible structures in the X-ray images. Further details concerning the age-control are given in [14], [15]. The cores were sliced at 1 cm intervals and, to avoid contamination between layers, the outer few millimetres were discarded from each layer. The core layers were individually placed in sealed plastic bags and kept at 4°C in the dark until further processing.

Sediment samples were rinsed and the living dinoflagellate cyst fraction was recovered by density separation [32]. While keeping the sediment fraction cool, individual *Pentapharsodinium dalei* cysts were isolated under the light microscope with micropipettes and set to germinate in 96-micro well plates filled with TL medium (see below) of salinity 25. After cyst germination, individual vegetative cells were isolated to establish clonal strains. The strains were kept in TL medium (salinity ∼23) under a light regime of ∼60 µmol photons m^−1^ s^−1^ in a light:dark cycle of 16∶8 at 15°C (temperature regulated room). From the total culture collection (>190 strains), we randomly picked six strains from three discrete layers of core K3: Layer 1 dated to 2006, Layer 2 from 21 cm depth, dated to 1960±5; Layer 3 from 34 cm depth dated to 1922±12 to be included in the growth rate experiment [15].

The 18 strains were grown at salinity 15 and 30 in triplicate 70 ml-polycarbonate flasks illuminated from below (total of 108 flasks), with an irradiance of 150 µmol photons m^−2^ s^−1^, and a light:dark cycle of 16∶8 h. All strains were acclimated to the experimental salinity and irradiance for 17 days prior to the start of the experiment. From the first day of acclimation, salinity was adjusted from the initial 23 in steps of ∼5 d^−1^. Thus, experimental salinities (15 and 30) were achieved 15 days before the start of the experiment. After the acclimation period, 300 exponentially growing cells ml^−1^ from each stock (strain and salinity) were inoculated in fresh TL medium and grown for three days before the first sampling.

TL medium is a standard enriched phytoplankton culture medium containing L1 trace elements [33], soil extract, and vitamins [34]. Nitrogen is added in the form of NaNO_3_ and Phosphorus in the form of Na_2_HPO_4_ 12 H_2_O, at final concentrations of 16.5 mgN l^−1^ and 1.7 mg P.l^−1^. Such concentrations of essential elements represent levels several times higher than those required to saturate growth rates or limit the biomass yield of phytoplankton cultures [35], [36]. However, pH will increase dramatically in unlimited-cultures with no air-exchange and become the growth-limiting factor before any nutrient becomes depleted. This is a consequence of a high photosynthetic activity, which will incorporate carbon into biomass faster than the supply from air-exchange and respiration, leading to the elevation of the pH. Therefore, when using confined flasks for culture experiments with high light and nutrient levels, it is important to keep track of pH and make sure that estimated growth rates are not affected by high pH limitation.

To estimate the exponential growth rate of the 18 strains in two salinities, we monitored cell concentrations and pH every 1–3 days, until the cultures had reached the stationary phase (due to elevated pH in the closed experimental flasks–up to 26 days). The sample volume (5 ml) was taken with a graduated pipette and replaced with fresh medium (pH = 7.5). The position of the flasks was changed randomly between samplings. Cell concentrations were determined manually, under a light microscope, by counting at least 300 acidic Lugol (1% final concentration)-fixed cells in Sedgewick-Rafter chambers. pH was measured immediately before sampling to the nearest 0.01 unit with a pH meter (Copenhagen pHM-83 Autocal). The pH sensor was calibrated on a daily basis using IUPAC buffers pH 7.0 and 10.0. The pH in the added fresh medium was adjusted (to 7.5) by adding 1 M HCl or NaOH. The concentration of dissolved inorganic carbon (DIC) in the fresh medium, measured using an infrared gas analyser (IRGA) and compared with a 2 mM standard, was 2.0 mM and 1.2 mM in 30 and 15 salinity, respectively.

Strain specific exponential growth rates (d^−1^) were determined in each successive sampling interval. Exponential growth rates µ (d^−1^) were calculated according to: µ = ln(x_t2_−x_t1_)/t_2_−t_1_, where x_t2_and x_t1_ is the cell concentration at end (t_2_) and start (t_1_) of the sampling interval, respectively. To obtain balanced exponential growth rate estimates, a minimum of 4 points (7 days) on the growth curve were included in the calculations. The end of the exponential phase was set as the first successive time interval yielding a significantly lower value than the average integrated growth rate from day 0–6 (Linear Model).

Upper pH tolerance limits for growth (the pH level where growth rate ≤0.0) were estimated from the parallel curves of cell-concentrations and pH as functions of time for each strain and salinity treatment. The upper pH limit was estimated as the pH level between the first sample interval yielding a growth rate not different from 0.0 d^−1^ (LM). For more details on upper pH tolerance limit calculations see: [30], [36], [37].

Diagnostic plots performed using the statistical software R [38], showed that the data on growth rates and cell concentration at stationary phase were normally distributed with equal variance, but the pH data were not (these were therefore rank-transformed). To investigate if the strains differed in growth rates and upper pH tolerance limits, we used a linear model (LM, corresponding to a one-way ANOVA) with growth rate/rank transformed pH limits as functions of strain (n = 18). Differences on growth at high and low salinity for each strain were tested using Students t-tests (n = 3). The effects of sediment layer (or cyst age) and salinity on the growth rates and upper pH tolerance limits were tested using a linear mixed effects model (LMEM) with strain as a random effect and both layer and salinity as fixed effects. By considering strain a random effect, the models account for variability due to strain. P-values for the fixed effects in the LMEMs were calculated using Maximum Likelihood Ratio tests (ML) comparing the model that contained the fixed effect with the reduced model without the fixed effect. Likelihood ratio tests were chi-square distributed. The LMEMs were conducted using the R-package lme4 [38]. Random effects were normally distributed for growth rates, rank transformed pH tolerance limits, and cell concentration at stationary phase, according to quantile-quantile plots of the predicted random effects.

## Results

**Figure 1:**
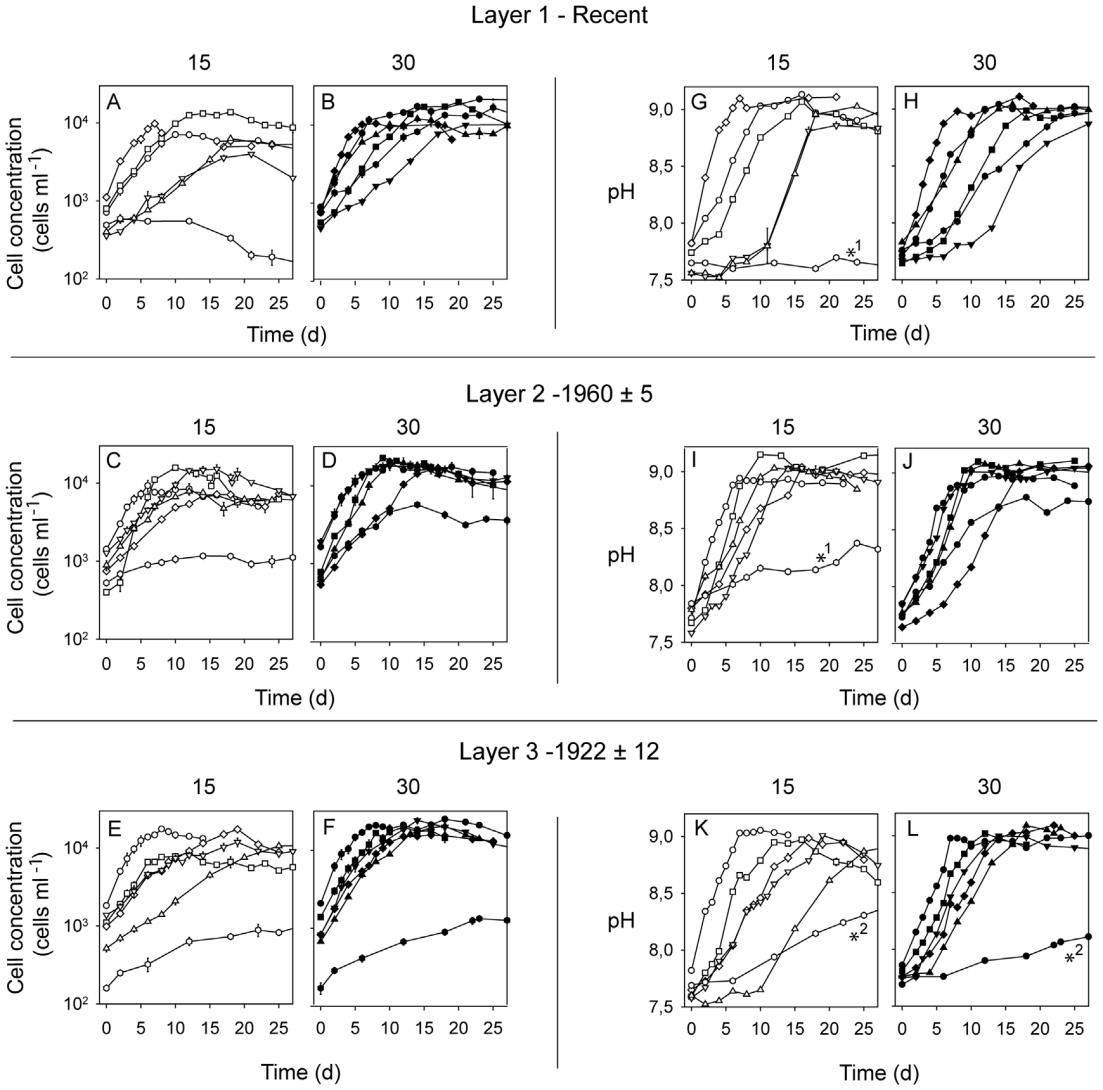
Cell concentration and pH as a function of time. Cell concentrations (A–F) and pH levels (G–L) are shown according to sediment core layer (age-depth) for the 18 Pentapharsodinium dalei strains tested at salinity 15 and 30. Points represent mean values of three replicates and bars represent s.e.m. (standard error of the mean). *1–strains for which upper pH limits could not be determined (see Results section); *2–strain for which upper pH limits were reached after 65 days.

Cell concentration increased exponentially as a function of time for all strains at both salinities for a period of 6–14 days (Figure 1 A–F) with the exception of one strain from layer 1 at salinity 15, where cell concentration increased initially, but cells ceased to divide after 5 days (Figure 1 A) (cyst formation was observed in this culture). No apparent lag-phases were observed and low variation between replicate flasks indicates that acclimation and balanced growth rates were successfully achieved prior to the start of sampling (time = 0). Following the increase in cell concentrations, pH increased as a function of time and reached maximum levels when the cultures ceased to grow (Figure 1 G–L). One strain from Layer 2 in the 15 salinity treatment stopped growing after 12–15 days of exponential growth and it was therefore not possible to determine its upper pH limit for growth (Figure 1 C). The mean cell concentration for all strains in the stationary phase was 15904±1484 cells ml^−1^ at salinity 30 and 8735±988 cells ml^−1^ at salinity 15. Tolerance limits for high pH varied little between the strains (overall range 8.7–9.1, Table S1) with an overall mean and median of 9.0 (Figure 1 G–L). Due to its low growth rate, one strain from Layer 3 reached the stationary growth phase and upper pH limit after 65 days (Figure 1 E–F, K–L, first 27 days shown).

Growth rates differed significantly among strains (LM, F = 24.32, p<<0.001, df = 18) and ranged from 0.02–0.65 d^−1^ with an overall mean of 0.31 d^−1^+−0.03 (Table 1). When comparing the growth rate at salinity 15 and 30 for each strain, 3 strains from layer 1; 2 from Layer 2; and 1 from Layer 3 showed statistically significant differences (Table 1).

**Table 1:** Observed growth rates for the 18 strains tested at salinity 15 and 30 with standard error.

Sediment core layer	Strain nr.	Salinity 15		Salinity 30		
		Growth rate (d^−1^)	Std. error (d^−1^)	Growth rate (d^−1^)	Std. error	p<0.05
Layer 1 (Recent)	1	0,33	0,06	0,32	0,06	
	2	0,15	0,05	0,28	0,06	*
	3	0,22	0,06	0,20	0,06	
	4	0,29	0,05	0,42	0,06	
	5	0,48	0,06	0,60	0,07	*
	6	0,02	0,05	0,22	0,06	*
	Mean	**0,25**	**0,06**	**0,34**	**0,06**	
	Range	**0,46**	**0,01**	**0,40**	**0,01**	
	CV (%)	**63**		**44**		
Layer 2 (1960±5)	7	0,43	0,06	0,41	0,06	
	8	0,65	0,05	0,45	0,06	*
	9	0,29	0,06	0,36	0,06	
	10	0,22	0,05	0,28	0,06	
	11	0,26	0,06	0,46	0,06	*
	12	0,11	0,06	0,23	0,06	
	Mean	**0,33**	**0,06**	**0,365**	**0,06**	
	Range	**0,54**	**0,01**	**0,23**	**0**	
	C.V.	**58**		**26**		
Layer 3 (1922±12)	13	0,38	0,04	0,36	0,04	
	14	0,35	0,06	0,37	0,05	
	15	0,29	0,06	0,35	0,06	
	16	0,24	0,06	0,35	0,06	
	17	0,16	0,06	0,17	0,07	
	18	0,18	0,05	0,37	0,07	*
	Mean	**0,26**	**0,05**	**0,33**	**0,06**	
	Range	**0,22**	**0,02**	**0,20**	**0,03**	
	C.V.	**34**		**20**		

The mean, range, and coefficient of variation (CV) are also provided for each layer (n = 18). * Student's t-tests were applied to test if there was a significant difference in growth rates at high and low salinity (5% significance level) (n = 3).

**Figure 2:**
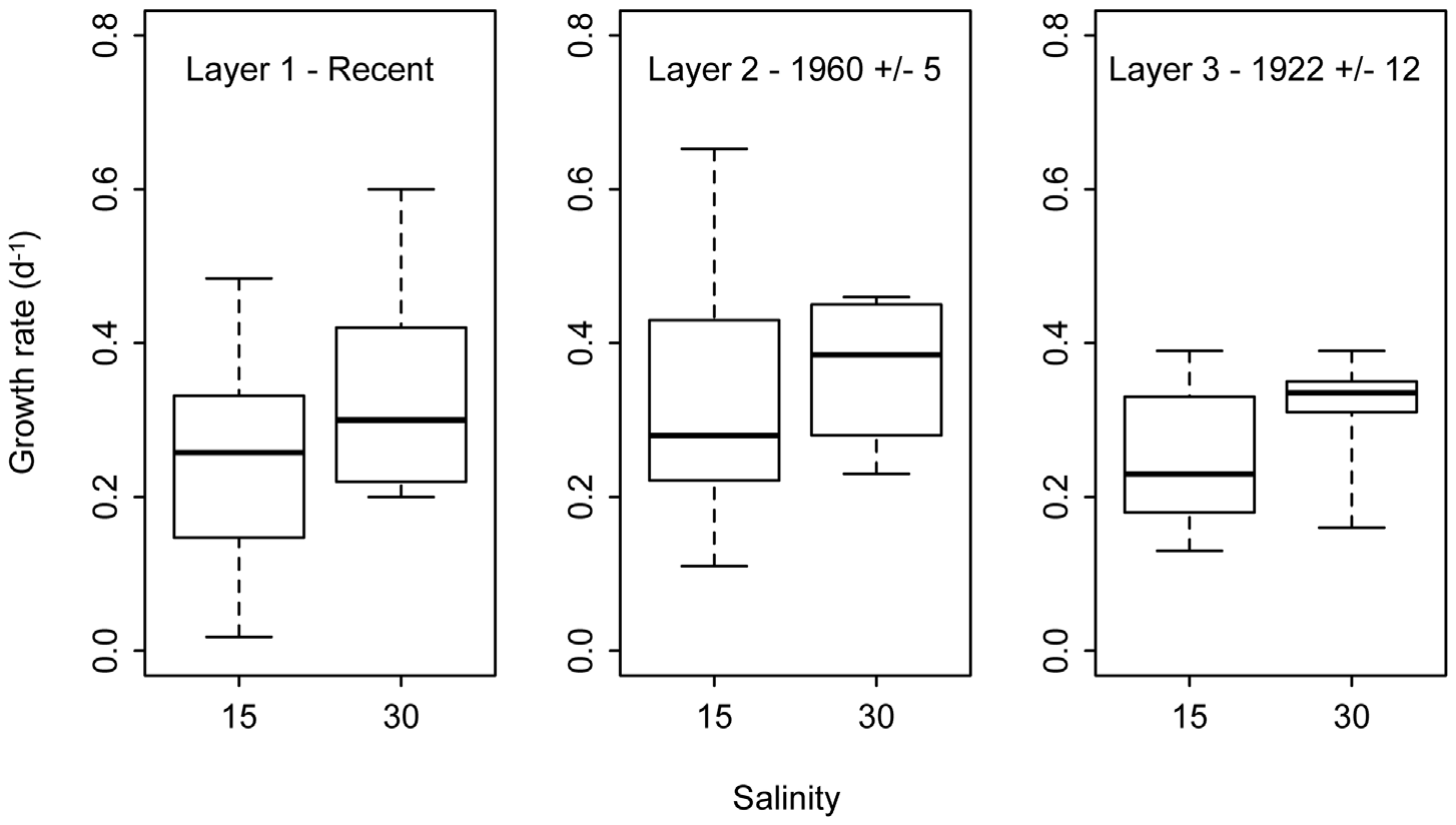
Growth performance of Pentapharsodinium dalei strains according to sediment core layer and salinity level. Boxplots show range (whiskers), median (bold line), and interquartile range (box height) for growth rates at salinity 15 and 30 for the three sediment core layers.

Overall, the mean growth rate was significantly higher at salinity 30 (0.35±0.03 d^−1^) than at 15 (0.21 d^−1^±0.01 d^−1^) (LMEM, X^2^ = 26.77, p<0.001, df = 1) (Table 2, Model 1). Growth rates for strains in the 15 salinity treatment were more variable, as shown by the higher coefficients of variation (Table 1), ranges and interquartile ranges (Figure 2). Median growth rates were significantly higher at salinity 30 than 15, although these differed less for layer 1 (Figure 2). When comparing the different layers, there was no statistically significant effect of time (cyst age) on growth rates at high and low salinity (LMEM, X^2^ = 0.825, p = 0.66, df = 2) (Table 2).

**Table 2:** Summary statistics for the applied Linear Mixed Effects Models (LMEM) of growth rate, median pH tolerance limits, and cell concentration at stationary phase as functions of cyst age/sediment core layer and salinity (fixed effects) with strain as a random effect.

Model 1: Effect of cyst age (layer) and salinity on growth rate			
Fixed effects	X^2^	df	p-value
Cyst age/layer	0.825	2	0.66
Salinity	26.77	1	***

Model 2: Effect of salinity and cyst age/layer on median pH tolerance limits			
Fixed effects	X^2^	df	p-value
Cyst age/layer	1.96	2	0.38
Salinity	0.04	1	0.83

Model 3: Effect of salinity and cyst age/layer on cell concentration at stationary phase			
Fixed effects	X^2^	df	p-value
Cyst age/layer	0.92	2	0.63
Salinity	27.99	1	***

P-values were calculated using Maximum Likelihood Ratio tests (ML). *** indicate p-values<0.01.

Upper pH tolerance limits were not significantly affected by salinity or cyst age (Table 2, Model 2). However, cell concentrations at stationary growth phase were significantly higher in the 30 than the 15 salinity treatment (LMEM, X^2^ = 27.99, p<<0.01, df = 1; Table 2, Model 3). This finding is related to the buffer capacity of the 15 salinity medium (∼1.2 mM DIC) compared to the 30 salinity medium (∼2.0 mM DIC). In the lower-buffered medium, pH changes more rapidly and reaches pH tolerance limits before the higher-buffered 30 salinity medium. This shows that growth and biomass yield was not limited by DIC at stationary phase, but by the high pH itself (as we can assume no N, P, or trace elements limitation due to the characteristics of the culture medium used–details given in the materials and methods section).

## Discussion

Our work represents a novel approach to the study of climate- and environmental-driven changes in marine phytoplankton populations. The possibility of reviving resting stages formed during a ca. 100-year period has made it possible to experimentally trace back the ecophysiological variability of *Pentapharsodinium dalei* in Koljö Fjord, Sweden. Our data suggest that two distinct and ecologically significant traits, i.e. salinity response and upper pH tolerance limits for growth were stable for this species across the studied time period.

The ecology of marine protists, particularly phytoplankton species, has traditionally been studied in the laboratory. Many studies have attempted to define the responses of species to changing environmental parameters by experimentally testing single strains, and often strains which had been kept in the laboratory for years or even decades [11], [12], [29]. These studies implicitly assumed that the experimentally measured properties of laboratory strains were static and reflected those of the natural populations. There are two fundamental problems with this tradition [10]. Firstly, marine protist species often consist of physiologically and genetically differentiated strains and strain-specific variation in growth rates is well documented [39]. Secondly, ecophysiological properties (such as growth rate as a function of salinity) may change during long-term culturing in the stable laboratory environment [10]. Data for growth rates and upper pH tolerance limits for growth of multiple strains of the common dinoflagellate *Heterocapsa triquetra* suggest that such changes occurred over a 50-year period of laboratory maintenance [30].The strains used in our experiment were revived simultaneously from resting stages deposited in sediments dating back to 1922±12. This allowed us to overcome potential concerns of physiological changes having occurred due to long-term maintenance in the laboratory. To address the other concern (i.e. strain-specific variability in natural populations), we randomly picked six strains from each sediment layer. The established strains were kept under identical conditions and grown at an intermediate salinity of 23 before acclimation into the tested experimental conditions (salinity 15 and 30). Our experimental setup is thus rather conservative.

The fact that revived *P. dalei* strains from all three layers generally grew better at higher salinity suggests a high level of homogenization despite environmental change across the 100-year time period. This homogenization may be due to the fact that, at any given time, resting stages in the sediment represent a mixture of newly formed and older cysts, resulting in considerable generation overlap. Furthermore, dormant propagule banks may slowdown the rate of evolution, because they effectively maintain diversity and sequester a fraction of the gene pool from the influence of microevolutionary processes in each generation, according to studies of lake copepods [40], and also microbial communities [41]. In a Danish fjord, resting cells of the coastal diatom *Skeletonema marinoi* formed over a >150 years period and analysed with microsatellite markers, revealed a single and genetically uniform population, distinct from populations of the same species found in open waters just outside the fjord [16]. Although distinct *Skeletonema marinoi* populations co-exist in the surface waters of the fjord, the large propagule bank presumably established quickly after an historical founder event appears to successfully act as a buffer against new immigrants [16]. Our results support the idea that phytoplankton resting stage banks may act as buffers against rapid environmental change.

Growth rate variability was high for the strains revived from Layers 1 and 2, but a drop in variability was evident in Layer 3, which represents strains revived from the oldest sediments, estimated to be up to one-century old. This drop in variability is probably associated to a drop in viability with age, as the germination success of *P. dalei* cysts isolated from Layer 3 (5%) was considerably lower than for Layers 1 and 2 (28 and 61%, respectively). This drop in potentially viable cysts, germination success and variability after ca. 50 years of dormancy indicates an upper limit to the study of past populations using the sediment archive of this species in this particular environment. When studying traits for which the response to different environmental conditions must be measured in living organisms, cyst viability will determine how far back in time phenotypes can be reconstructed. However, “ancient” DNA retrieved directly from the cysts may reveal population changes at the genetic level further back in time. DNA has been retrieved from diapausing microcrustacean eggs in lake sediments dated to ca. 200 years [42], and we have successfully genotyped *P. dalei* cysts retrieved from Koljö Fjord sediments dated to ca. 100 years using microsatellite makers (unpublished data).

Our study revealed high intraspecific variability down to sediment layers estimated to be up to ca. 50 years-old, showing that the cyst bed of *P. dalei* is a repository of ecophysiological diversity. Long-lived resting stage banks have been recognized for a long time in terrestrial ecosystems (i.e. plant seed banks) and in lakes [41], [43], [44] as archives of both genetic (intrapopulacional) and ecological (interspecific) information, but remain less investigated in marine environments (with the exception of benthic resting eggs of copepods). In coastal areas, environmental conditions fluctuate largely on a seasonal basis, and may also change markedly at multi-year scales (e.g. events of bottom water exchange and oxygenation in Koljö Fjord). Coastal phytoplankton populations are typically discontinuous, occurring at high concentrations (blooms) only during limited periods of favourable conditions. Additionally to being a product of sexual reproduction (in most cases), it seems likely that long-lived dinoflagellate cysts have been selected for as an effective bet-hedging strategy in these fluctuating environments [45].

Our finding that *P. dalei* from all 3 layers grows better at high (30) rather than low (15) salinity indicates that salinity alone fails to explain the cyst record of the species in Koljö Fjord. Maximum abundances of *P. dalei* cysts are found in sediments dated to 1930–1980, when average sea-surface salinity is reduced. Harland and co-authors [20] investigated the dinoflagellate cyst record of Koljö Fjord, and suggested that nutrient availability in the surface waters of the fjord during spring and summer, and the establishment of a well-stratified and stable water column are the main factors determining changes in the cyst record. *Pentapharsodinium dalei* is found in coastal areas associated with high productive and well-stratified waters [23], [26], [27]. The period corresponding to the highest *P. dalei* abundances in Koljö fjord coincides with the beginning of anthropogenic nutrient loading (cultural eutrophication) in many coastal areas along the Swedish coast. However, the area surrounding the fjord has no extensive farming, no large urban centers, and no industry. Furthermore, nutrient measurements in the water column show no significant increase since the 1960's, and the organic carbon content of the sediments is higher for the periods 1820–1930 and post-1980, and lower for the period 1930–1980 (when *P. dalei* is most abundant) [20]. The predominantly negative NAO phase of 1930–1980 is associated with the occurrence of frequent easterly and north-easterly winds, increasing offshore upwelling. This leads to higher bottom water salinity in the fjord, the formation of a strong pycnocline, and increased water column stability. Rather than responding directly to salinity, it is likely that the *P. dalei* fjord population has, from 1930–1980, benefited from a generally increased stability of the water column, and the nutrient supply brought to the surface by terrestrial runoff in the spring.

Our study indicates that the marked changes in salinity experienced in Koljö Fjord over the past 100 years did not induce a significant alteration in the salinity response of *Pentapharsodinium dalei* populations, nor a change in upper pH tolerance limits for growth. The inferred stability suggests that the response of modern populations may be extrapolated to assemblages of morphospecies back through time despite changes in the environment-a fundamental principle in (palaeo)environmental studies. However, we cannot rule out the possibility that other traits may be more susceptible to evolutionary change as selection pressures may differ considerably between environmental factors. There is also a possibility that subtle changes in populations may become apparent if the number of studied strains is increased or when natural populations are investigated. In a microcosm experiment with *Skeletonema marinoi* abiotic stressors were reported to affect differently monocultures and mixtures of genetically distinct strains [46]. By taking advantage of future experimental advances (e.g. flow cytometry, fluorometry and gene-expression) further studies in the presented frame-work including more strains may provide valuable insights into the responses of natural populations to environmental change and how these may affect ecosystem functioning. One promising approach is the application of high-throughput sequencing techniques to study microevolutionary changes at the genome level linked to environmental change.

## Supporting Information

Table S1 Upper pH tolerance limits for growth for the 18 *Pentapharsodinium dalei* strains at salinity 15 and 30.
(DOCX)
