# Supplementary material for: Hundred Years of Environmental Change and Phytoplankton Ecophysiological Variability Archived in Coastal Sediments
Source: PLoS One. 2013 Apr 11;8(4):e61184. doi: 10.1371/journal.pone.0061184 (PMC3623915; doi:10.1371/journal.pone.0061184)
Supplement: Table S1 — Upper pH tolerance limits for growth for the 18 Pentapharsodinium dalei strains at salinity 15 and 30. (DOCX) [file pone.0061184.s001.docx]

**Table S1**

| Sediment core layer | Strain nr. | Salinity 15 | | Salinity 30 | |
| --- | --- | --- | --- | --- | --- |
|  |  | Upper pH limit | Std. error | Upper pH limit | Std. error |
| Layer 1 (Recent) | 1 | 8,92 | 0,01 | 8,95 | 0,00 |
|  | 2 | 8,99 | 0,01 | 9,02 | 0,00 |
|  | 3 | 8,85 | 0,02 | 8,87 | 0,00 |
|  | 4 | 9,06 | 0,01 | 9,05 | 0,01 |
|  | 5 | 9,03 | 0,00 | 9,02 | 0,00 |
|  | 6 | - | - | 8,95 | 0,01 |
|  | 7 | 8,99 | 0,00 | 9,06 | 0,01 |
| Layer 2  (1960±5) | 8 | 8,92 | 0,01 | 8,93 | 0,00 |
|  | 9 | 9,02 | 0,01 | 9,06 | 0,00 |
|  | 10 | 9,01 | 0,05 | 9,01 | 0,00 |
|  | 11 | 9,01 | 0,00 | 9,04 | 0,00 |
|  | 12 | - | - | 8,74 | 0,02 |
|  | 13 | 9,05 | 0,00 | 8,97 | 0,01 |
| Layer 3  (1922±12) | 14 | 8,96 | 0,00 | 9,00 | 0,00 |
|  | 15 | 8,94 | 0,03 | 9,03 | 0,00 |
|  | 16 | 8,90 | 0,01 | 9,00 | 0,01 |
|  | 17 | 8,82 | 0,02 | 8,87 | 0,00 |
|  | 18 | 8,99 | 0,01 | 9,01 | 0,00 |
